# Supplementary material for: Real‐time detection of condensin‐driven DNA compaction reveals a multistep binding mechanism
Source: EMBO J. 2017 Nov 8;36(23):3448–57. doi: 10.15252/embj.201797596 (PMC5709735; doi:10.15252/embj.201797596)
Supplement: Supplementary file 1 — Appendix [file EMBJ-36-3448-s001.pdf]

Appendix

Contents

Supplemental methods

- Step-fitting analysis (includes Appendix Figure S1)
- Step validation (includes Appendix Figure S2)
- Response time of tethered bead (includes Appendix Figure S3)

Step fitting analysis

We used a user-independent step-finding algorithm, which has been previously described and abundantly used in many biophysical studies (Kerssemakers *et al*, 2006). Briefly, the method works as follows. With this method, the best step fit is performed based on least-square minimization. The same number of steps is applied to the same data, but is then deliberately misplaced, such that fitted step locations are positioned at places away from the steps (a so-called 'counter-fit'). The more prominent the steps are in a trace, the worse this counter fit is (i.e. the higher the chi-squared). For a featureless trace, the best and worst fit are not very different. Therefore, the ratio between the chi-squared value of fit and counter-fit reaches a maximum at the most likely number of steps, whereas this ratio is 1 if there are no steps. We refer to this ratio as the S-value (Kerssemakers *et al*, 2006). In our analysis, we reject the fit if this S-value is below 1.15. Thus, as was shown before, by optimizing the S-value, an optimal fit can be found as a function of the number of fitted steps.

For complicated step traces, this procedure tends to underestimate the number of steps. Therefore, we performed a two-pass algorithm. To evaluate the variation of step sizes in an objective manner, we improved the implementation of this algorithm to allow for hands-off, batch style analysis. We followed an automated, three-phase analysis workflow:

1. We performed a "major step fit" as described above. Based on the individual errors for these fitted steps, we determined an "error-threshold".
2. This primary step fit was subtracted from the data. The residue may still contain smaller steps.
3. A "minor step fit" was performed on the residue with the same rejection criteria as for the first round, with however the addition that the step errors needed to be below the "error-threshold".
4. Finally, all accepted step locations were combined to build the final fit. All these steps thus have similar error margins.

Appendix Figure S1 presents an example of the step fitting protocol.

We confirmed the validity of the step fit by evaluating the S-value. For example, as described (Kerssemakers *et al*, 2006), for perfect steps of 170 nm with noise comparable to our experiments (102 ± 28 nm, one sigma, mean ± SD), the maximum expected S-value is (1+(170/(2*102))^2)=1.7. For our experimental data, we found a typical ratio maximum of around 1.5 ± 0.3 (mean ± SD), in good agreement with this estimate. We conclude from this that the steps we detect are indeed well-defined, i.e., consisting of sharp transitions and flat plateaus and not just a product of false-positive fits of noise.

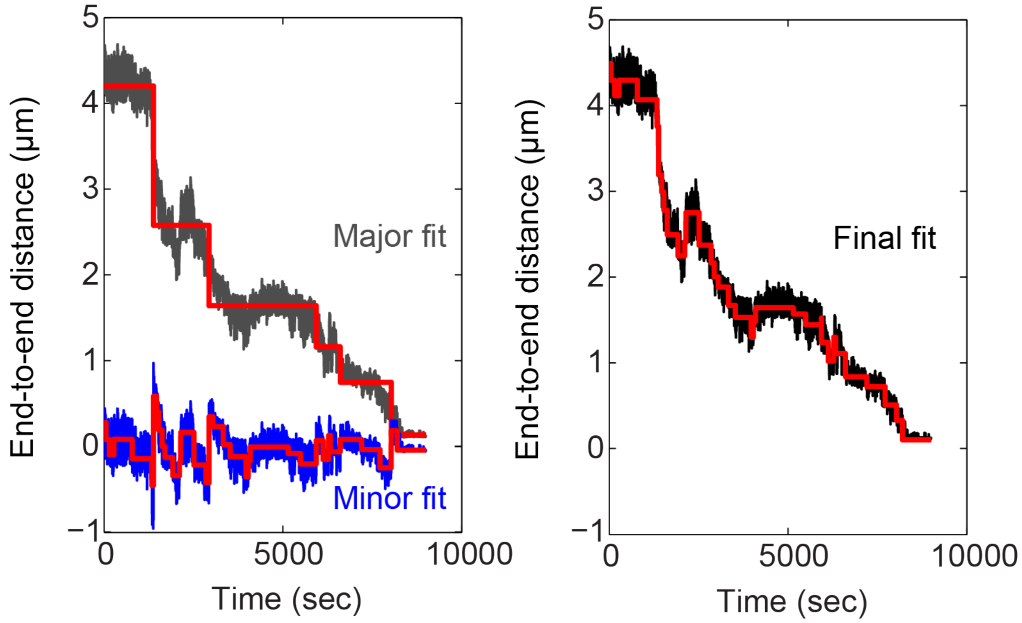

Appendix Figure S1: Principle of multi-pass automated step fitting analysis.

First, a major step fit (upper red curve) is done on the data (left panel, gray). This fit is subtracted from the original data. Next, a minor step fit is done on the residue (left panel, blue). Accepted step fits are combined in a final fit (right panel).

Step validation

When we used the step-finder as described, we obtained a wide distribution of steps (Figure 4B). To check how reliable these steps were, we set up a test routine to sporadically inject artificial steps of user-defined size into our experimental data traces. We subsequently evaluated how well our step-finding algorithm was able to detect these. For this validation routine, we used the experimental curves at standard conditions (0.75pN, 1mM ATP, 8.6nM condensin) as a representative set of 20 curves, which included the typical noise and occasional spikes. To preserve the overall compaction rate, each positive step was followed by a negative step. We added multiple (~4) up-down pairs per curve (example shown in Appendix Figure S2A). For building statistics, each curve was used 20 times, each time with a different selection of injection steps of defined size. The size for the injected steps was randomly picked from a 0-1500nm step-size range, thus covering at least the range of originally detected steps.

Consistent with our conservative choices in setting up the step-extraction protocol, we find that only a finite fraction of the steps is detected. For larger-size steps (roughly larger than 500nm), most steps are detected, but, by contrast, most small steps (~100nm) stay undetected (Appendix Figure S2BC).

This result has important consequences for what can be concluded from our (and other) magnetic tweezer experiments under these low-force conditions, where tweezers traces intrinsically exhibit large noise. Most noteworthy is that small steps easily get drowned by noise or are obscured by neighboring steps. Accordingly, we cannot make a firm statement on a characteristic (small) step size associated with the condensin-induced DNA condensation process. At the same time, larger steps are detected reliably, and we can conclude that these indeed occur in the DNA condensation traces. Overall, we conclude that the measurement traces signal a very broad distribution of step sizes, including remarkably large steps.

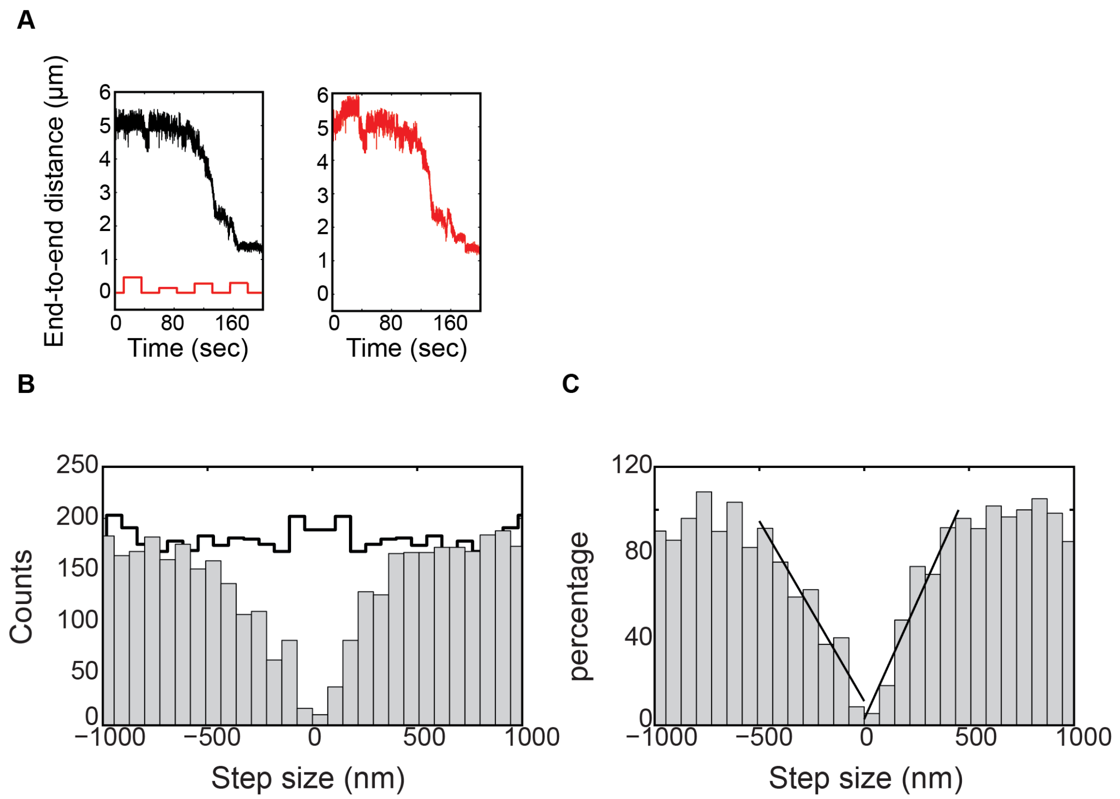

Appendix Figure S2: Step validation.

A) On the left side, an experimental trace is shown (black) as well as the “fake” steps (red) that were added to the experimental trace. The right panel shows the combined experimental curve plus injected steps.

B) Histogram showing the distribution of returned steps (light grey) from all injected steps. For reference, the input distribution is shown with a line in the background.

C) Histogram of return percentage per step size. Line is a phenomenological fit of the linear regime to the data, which was used for correcting the original step distribution histogram in Figure 4b in the main text, see inset to Fig.4b.

Response time of the tethered bead

In our magnetic tweezers experiment, the magnetic bead cannot respond infinitely fast to an instant (condensation) step, since it is slowed down by the drag associated with moving the bead through liquid. Here, we follow a simplified approach to obtain a ballpark estimate of the response time of the bead. This situation is reminiscent of the force-switch experiments performed previously (Crut *et al*, 2007).

We simplify the stretched DNA at an extension *e* as a linear spring with spring constant *k(e).* We also assume a mass-less, heavily overdamped system. For determining *k*, we assume the DNA behaves as a worm-like-chain. We expect the DNA to be stiffer after compaction, as the contour length is decreasing (Appendix Figure S3).

We start out with a stretched tether, where the magnetic force is balanced by the entropic stretching force of the DNA. Then, we assume that a condensation step instantly shortens the DNA by 200nm. As the bead is initially still in the same position, the DNA is stretched by the same amount of 200nm. During displacement of the bead following an instant shortening of the DNA, the drag force experienced by the bead is balanced with the DNA spring force:

$$F_{drag} + F_{spring} = 0 \text{ , or } \gamma \frac{dx}{dt} + \kappa x = 0$$

with $6\pi\eta r$ characterizing the Stokes drag of the bead, η the viscosity of water, and r the radius of the bead (0.5μm). The bead will therefore move according to

$$x(t) = x_0 e^{-t/\tau}$$

with $\tau = \frac{\gamma}{\kappa}$ the response time of the bead.

We evaluated the response time for three points: near the initial bare DNA extension at the applied pulling force (*F*=0.3pN, *e*=4.7μm), at halfway compaction (*e*=2.0μm), and at nearly complete compaction (e=0.6μm) (see Appendix Figure S3). This yields values of *k* = $0.2145 * 10^{-6}$ to $0.8005 * 10^{6}$, leading to response times of τ = 100 to 400ms, respectively. This estimate is consistent with experimental measurements (Crut *et al*, 2007).

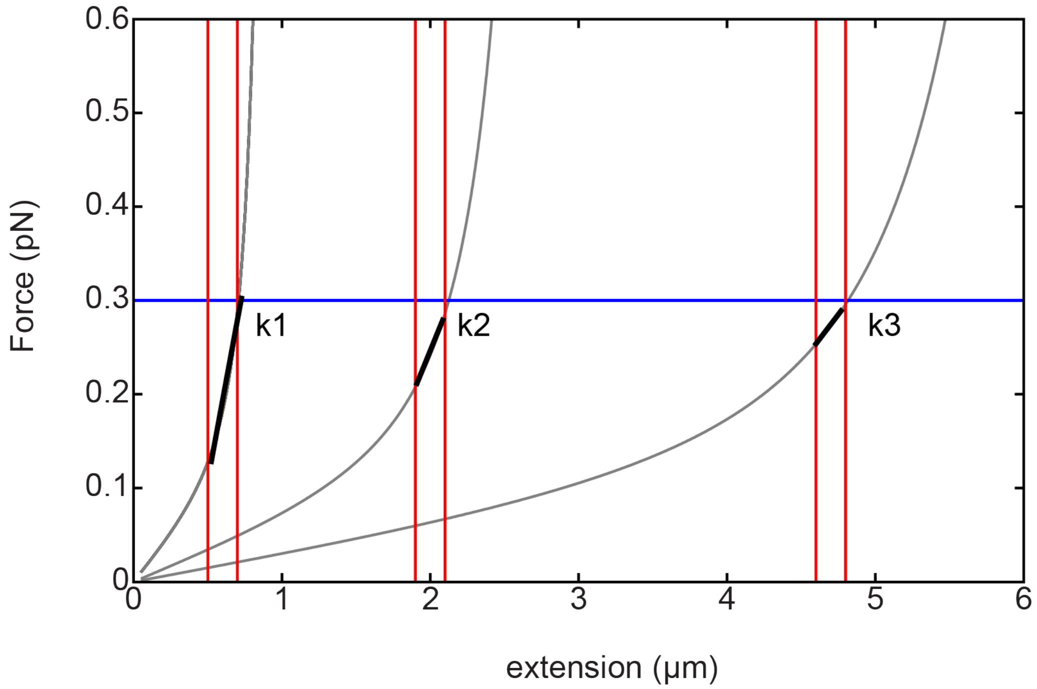

Appendix Figure S3: Determining the spring constant of the DNA at three compaction states.

Supplementary references

Use the "Insert Citation" button to add citations to this document.
