# Supplementary material for: Real‐time detection of condensin‐driven DNA compaction reveals a multistep binding mechanism
Source: EMBO J. 2017 Nov 8;36(23):3448–57. doi: 10.15252/embj.201797596 (PMC5709735; doi:10.15252/embj.201797596)
Supplement: Supplementary file 2 — Expanded View Figures PDF [file EMBJ-36-3448-s002.pdf]

Expanded View Figures

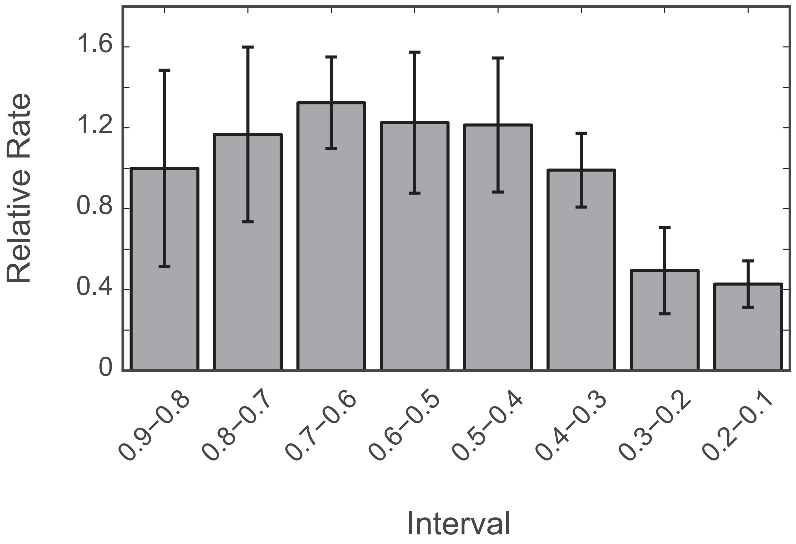

Figure EV1. Relative average rate as compaction progresses.

The compaction rate is relatively steady over the course of a condensation experiment, but slows down towards the end. Rate depicted relative to the rate in the interval of 0.9–0.8 of the original end-to-end distance as measured in the pre-measurement. Error bars are SEM, $N = 16$.

© 2017 The Authors

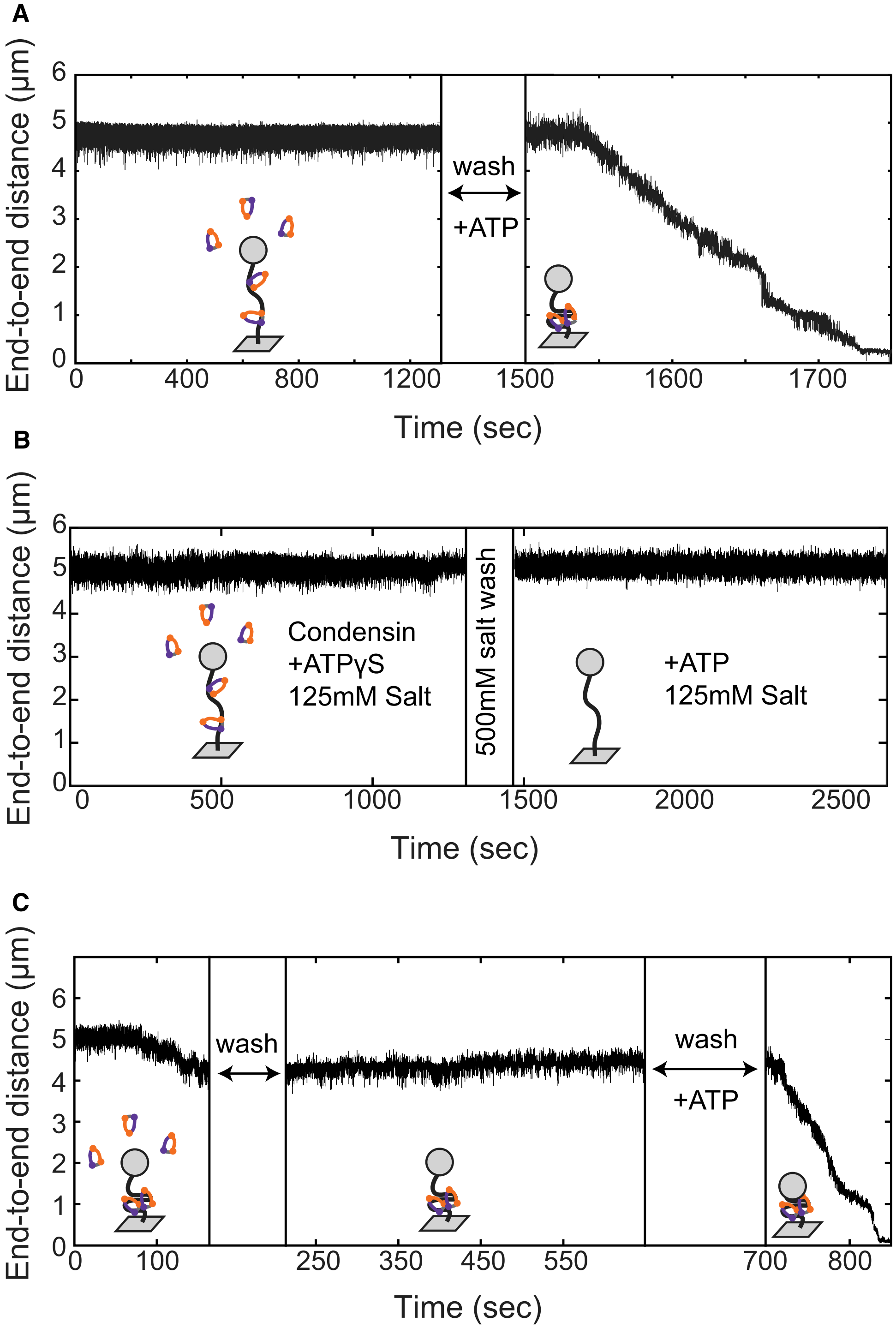

Figure EV2. Condensin does not compact DNA in the absence of hydrolysable ATP or at high salt.

A Example trace of DNA when condensin is added in the absence of ATP. After a 20-min incubation, no condensation is observed. After washing the flow cell with buffer with no additional protein (t = 1,300), ATP is added (t = 1,500) and compaction is observed, indicating protein was bound in the absence of ATP.

B At t = 0, condensin and ATPγS are added; no compaction is observed. The flow cell is then washed with high salt (500 mM). Next, ATP and physiological salt are added, and no compaction is observed. This indicates that ATP hydrolysis is necessary to achieve salt-resistant binding.

C At t = 0, condensin and ATP are added and compaction is initiated. We interrupt ongoing DNA compaction by flushing with buffer without ATP (t = 150). The compacted DNA remains compacted after washing with buffer without extra protein or ATP. Addition of ATP is necessary for compaction to proceed (t = 700).

© 2017 The Authors

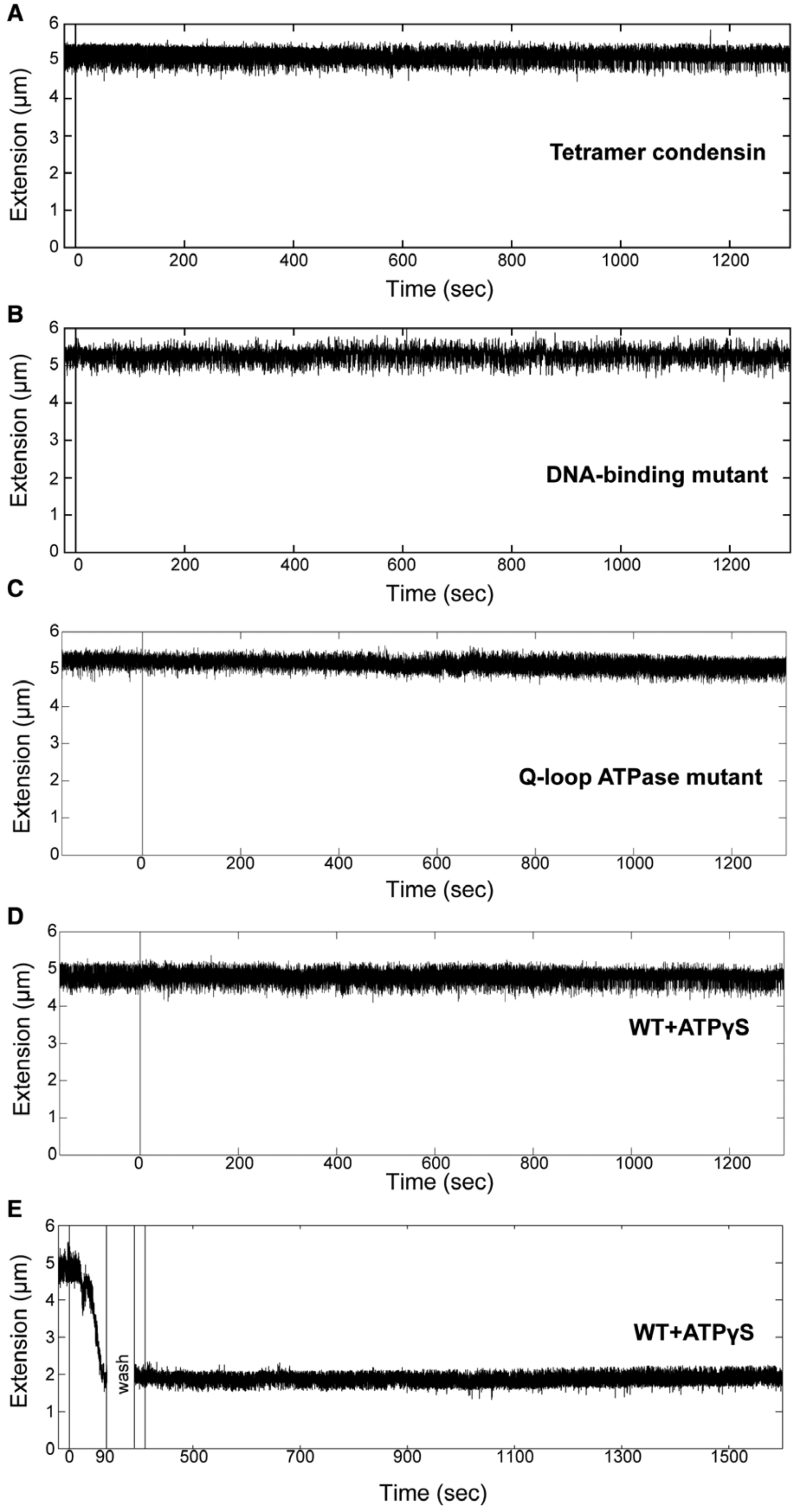

Figure EV3. Condensin tetramer, DNA binding mutant condensin, ATPase mutant condensin and wild-type protein + ATPγS do not compact DNA.

- A Example trace showing that tetramer (lacking Ycg1) condensin does not compact DNA.
- B Example trace showing condensin with mutations in the DNA-binding domain does not compact DNA.
- C Representative trace showing that the Q-loop condensin mutant does not compact DNA ($N = 12$). Protein is added at $t = 0$.
- D Representative trace showing that the wild-type condensin protein does not compact in the presence of ATPγS ($N = 11$). Protein + ATPγS is added at $t = 0$.
- E Representative trace showing that ATP hydrolysis is necessary for compaction ($N = 6$). At $t = 0$, protein and ATP is added as normal. After half compaction ($t = 90$), the flow cell is washed. ATPγS is added at $t = 400$. Compaction is unable to continue in the presence of ATPγS.

© 2017 The Authors

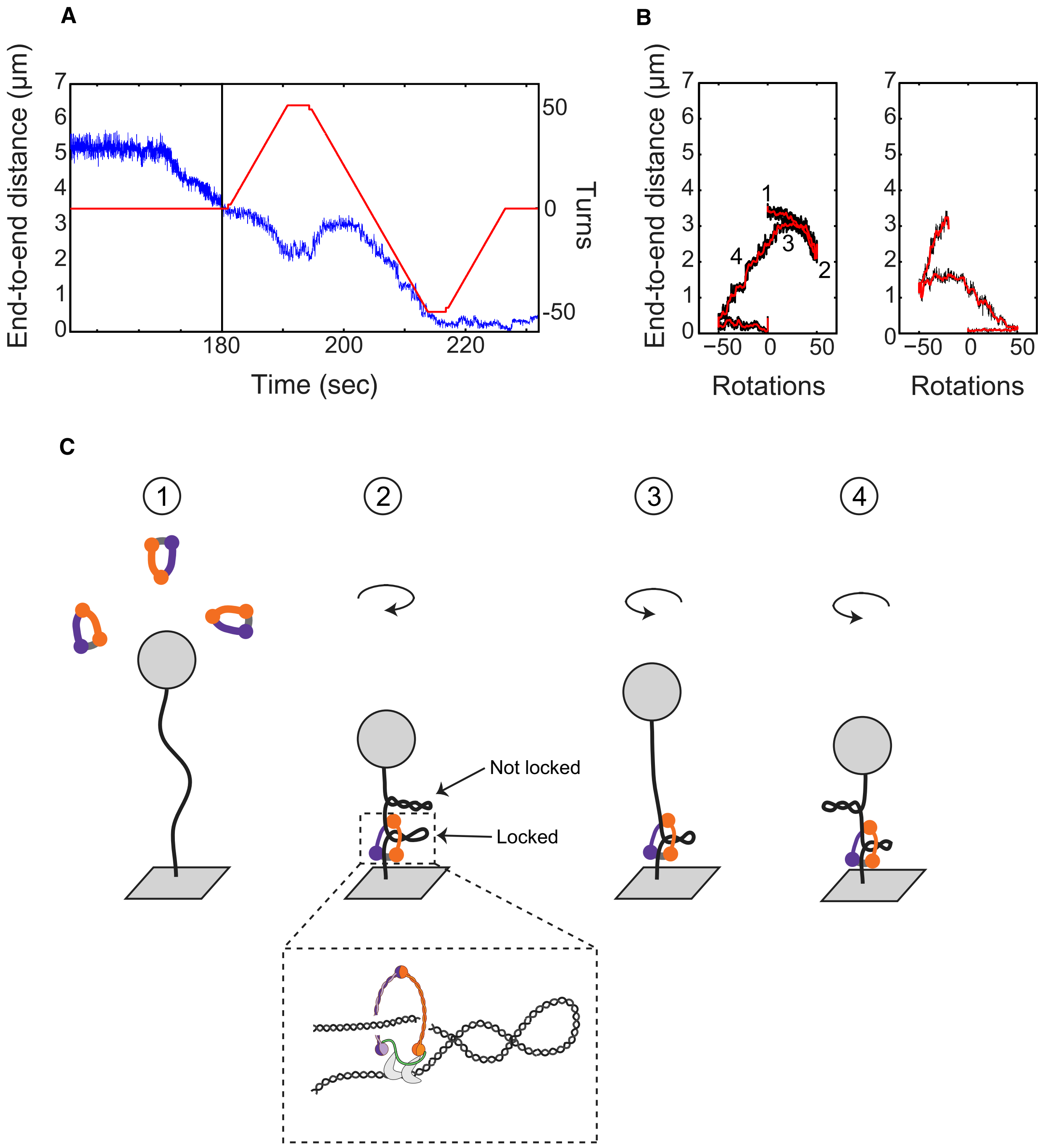

Figure EV4. Condensin shifts rotation curves.

A Example of experiment with the compaction trace shown in blue and the rotations shown in red. Tether was compacted by condensin as usual. After about 50% condensation (at time = 180), 50 positive rotations were applied. Then, the magnet was turned to −50 and back to 0.

B Rotation curves of condensed DNA at 0.75 pN. Left curve shows end-to-end length as a function of rotation, corresponding to the trace in panel (A) (after time = 180). Right curve shows similar experiment, only here negative turns were applied first. Numbers correspond to the states depicted in (C).

C Proposed model for condensation curve shifts in condensed molecules. We speculate that condensin is able to lock plectonemes. First, the DNA is "relaxed", as no rotations are introduced yet. Second, the end-to-end length has decreased because 50 turns were absorbed (either positive or negative). Third, the turns are released as the magnet is rotating back to zero, but because condensin has "locked" some plectonemes, the end-to-end length of the DNA cannot be fully recovered. Instead, the rest of the DNA is essentially relaxed before reaching 0 turns. Therefore, the DNA starts absorbing the rotations, and end-to-end length is decreased again. In the shown examples, this shifts the highest point of the rotation curve from 0 to around 25, indicating that condensin has locked about 25 turns into a plectoneme. This process happens regardless of the initial rotation direction, suggesting that condensin can lock both positive and negative supercoils.

 © 2017 The Authors

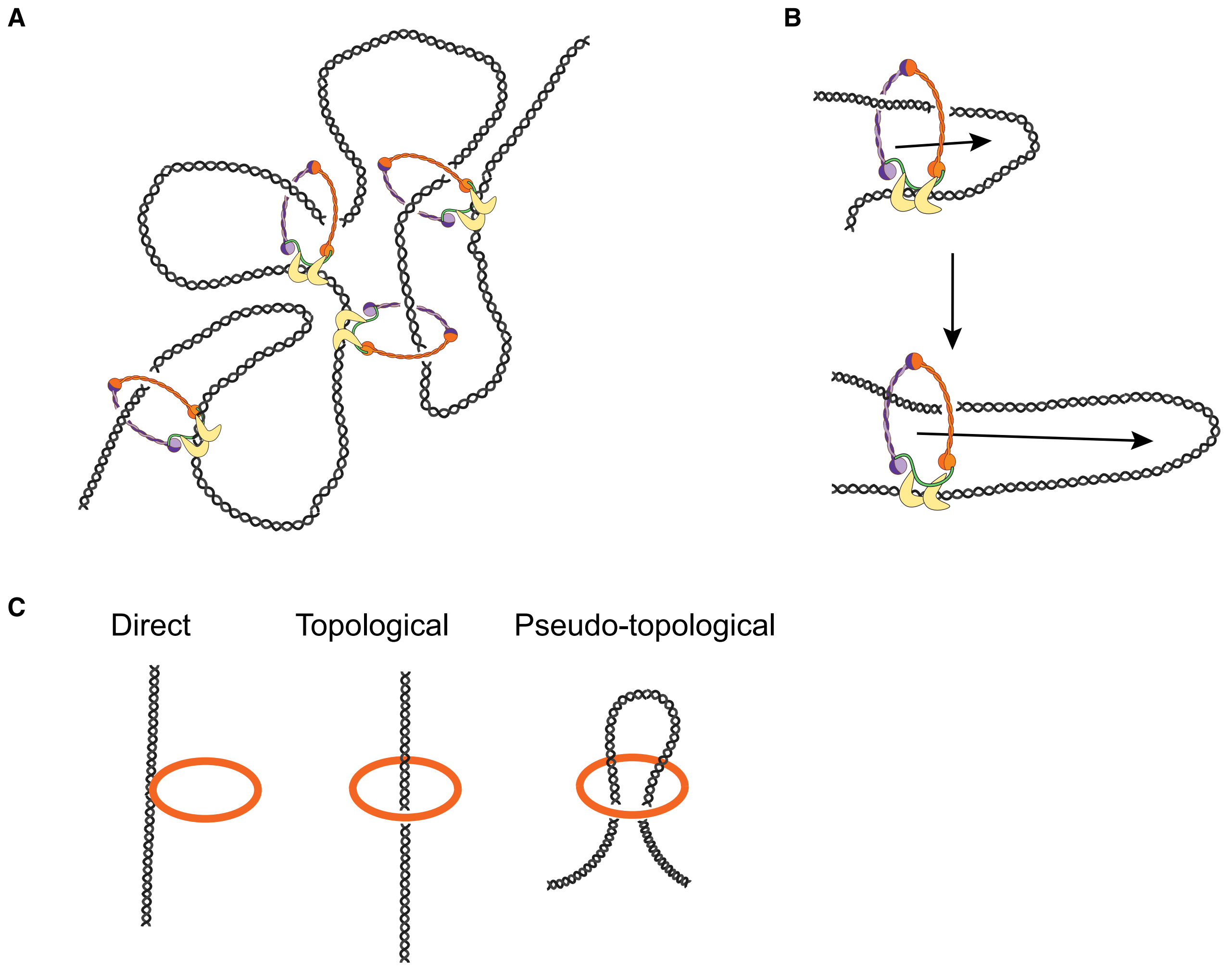

Figure EV5. Models for DNA compaction.

A The random cross-linking model.
B The loop extrusion model.
C Direct, topological and pseudo-topological loading.

© 2017 The Authors
